# Antilipase activities of cultivated peppermint and rosemary essential oils: in vitro and in silico studies

**DOI:** 10.55730/1300-0152.2725

**Published:** 2025-01-14

**Authors:** Khadidja BENGANA, Talia SERSEG, Khedidja BENAROUS, Arif MERMER, Yakup ŞİRİN, Alaeddine KAOUKA

**Affiliations:** 1 Laboratory of Fundamental Sciences, Faculty of Sciences, University of Amar Telidji, Laghouat, Algeria; 2 Department of Biology, Faculty of Sciences, University of Amar Telidji, Laghouat, Algeria; 3 Applied Sciences and Didactics Laboratory, Higher Normal School, Laghouat, Algeria; 4 Department of Biotechnology, Faculty of Health Sciences, University of Health Sciences, İstanbul, Turkiye; 5 Experimental Medicine Application and Research Center, Validebağ Research Park, Faculty of Health Sciences, University of Health Sciences, İstanbul, Turkiye; 6 Department of Pharmacy, Faculty of Health Sciences, University of Health Sciences, İstanbul, Turkiye; 7 Research and Development Center, Semas Food Ind. Trade Co. Ltd., Ankara, Turkiye

**Keywords:** Essential oil, lipase, molecular docking, peppermint, obesity, rosemary

## Abstract

**Background/aim:**

The growing interest in essential oils clearly indicates the power of nature and aligns with our increasing need to find therapeutic solutions in the natural world. This study aimed to investigate the inhibitory effects of the essential oils of *Mentha × piperita* and *Salvia rosmarinus*, harvested from the Laghouat region of Algeria, against *Candida rugosa* lipase (CRL) and pancreatic lipase through both in vitro and in silico studies.

**Materials and methods:**

Essential oils were extracted via hydrodistillation and analyzed using gas chromatography–mass spectrometry and spectrophotometry. Their antilipase activities were assessed using an inhibition assay, and molecular docking was performed with AutoDock Vina to explore interactions between essential oil compounds and lipase enzymes.

**Results:**

Spectrophotometric analysis demonstrated significant inhibitory activity for each essential oil against CRL lipase, with IC_50_ values of 0.56 ± 0.005 and 0.69 ± 0.008 mg/mL for peppermint and rosemary oils, respectively. These results were satisfactory in comparison to those achieved with orlistat. Molecular docking studies revealed the mechanisms of major compounds in each essential oil, demonstrating that these compounds inhibited CRL (PDB ID: 1CRL) and pancreatic lipase (PDB ID: 1LPB) with repeated hydrophobic interactions. The interactions were observed to be consistent with His449, Gly123, Gly124, Phe344, and Ser152 for many molecules.

**Conclusion:**

This study highlights opportunities for essential oils and their bioactive components to be utilized as adjuvants in the management of obesity and other lipase-related disorders.

## 1. Introduction

Natural products have gained considerable attention as a rich source of biologically active compounds. There is an increasing need to incorporate these natural substances into pharmaceutical research to develop effective therapies with fewer side effects (Clark, 1996; Amaral-Machado et al., 2020; Sauter, 2020; Zhang et al., 2020; Najmi et al., 2022). Among these compounds, many have shown potential in treating various medical conditions, including obesity. Obesity constitutes a global health threat (Blüher, 2019; Kopelman, 2000; Safaei et al., 2021). According to the World Health Organization, in 2022, 2.5 billion adults aged 18 and older were classified as overweight, with 890 million of them considered obese.^1^ This alarming figure continues to rise, and if left unchecked, the prevalence of obesity will keep increasing in the coming years. Obesity is not only a major health concern but also a contributor to several serious conditions, such as type 2 diabetes (Kopelman, 2000; Safaei et al., 2021), liver disease, and cardiovascular complications (Kopelman, 2000; Lopez-Jimenez et al., 2022). It also imposes daily challenges on affected individuals, leading to increased sedentary behavior, which negatively impacts the global economy (Hammond and Levine, 2010; Okunogbe et al., 2021). One of the key contributors to obesity is the excessive activity of the enzyme lipase (Lunagariya et al., 2014; Rocha et al., 2023), which breaks down triglycerides into glycerol and fatty acids that are stored as triglycerides in adipocytes (Miyake, 2001; Birari and Bhutani, 2007; Kumar and Chauhan, 2021; Rocha et al., 2023). The prolonged accumulation of adipose tissue leads to weight gain and, ultimately, obesity.

Peppermint (*Mentha × piperita*) is a member of the family Lamiaceae renowned for its distinctive flavor and medicinal attributes, which are primarily attributed to its chemical constituents. Since antiquity, the essential oil of peppermint has been utilized in medicine and disinfection and has served various other functions, including use as a deodorant. Peppermint oil is a well-known antioxidant that protects cells from damage caused by free radicals and has antiinflammatory, antibacterial, and antifungal properties (Burbott et al., 1983; McKay and Blumberg, 2006; Khalvandi et al., 2019).

Rosemary (*Salvia rosmarinus*) is a shrub species of the family Lamiaceae. The essential oil of rosemary is extensively utilized in traditional medicine and aromatherapy for its numerous health advantages. It contains compounds that improve memory and concentration; its inhalation is believed to augment cognitive performance, mitigate pain and muscular inflammation, and reduce respiratory issues such as congestion, asthma, sore throats, and colds. It possesses a calming antibacterial effect and enhances intestinal health (Soliman et al., 1994; Boutabia et al., 2016; Hannour et al., 2018; Lešnik et al., 2021; Annemer et al., 2022).

This study aimed to explore safe and effective solutions to obesity by inhibiting lipase activity using natural inhibitors derived from the essential oils of *M. × piperita* and *S. rosmarinus* sourced from the Laghouat region of Algeria. The chemical composition of peppermint and rosemary essential oils was determined using gas chromatography–mass spectrometry (CG-MS) and then the efficacy of these essential oils in inhibiting *Candida rugosa* lipase (CRL) was evaluated. Subsequently, binding affinities and interactions between the constituents of the oils and CRL and human lipase were investigated with molecular docking studies.

## 2. Materials and methods

### 2.1. Plant material

This study was carried out at the ETS Aquacol Laghouat aquacultural farm in Laghouat, Algeria (33°47′N, 2°50′E; elevation of 750 m). The province of Laghouat is bordered by Tiaret to the north, El Bayadh to the west, Ghardaia to the south, and Djelfa to the east. It is located in the heart of the Saharan Atlas range and extends across the steppe. The climate^2^ is continental, with extremely hot summers and freezing winters. Rainfall in the region is uneven, averaging 180 mm per year, with significant droughts in some years (Boumeddiene et al., 2023).^3^ The site has arid sandy soils with high water permeability and pH of 6.9.

We planted *M. × piperita* using seedlings received from Haffaci Ahmed, a member of the Professional Council of Aromatic and Medicinal Plants of Laghouat. We planted the seedlings in the field in the spring of 2022 in a location that receives direct sunlight for about 6–8 h each day, as peppermint thrives in sunny conditions, and we watered the plants three times a week. This watering schedule helped keep the soil consistently moist without becoming waterlogged. To preserve the plants from overharvesting and prevent desertification, we planted *S. rosmarinus* from cuttings collected in the El-Ghicha region of Laghouat (33°56′14″N, 2°08′54″E; elevation of 1207 m). Rosemary seedlings were planted in the spring of 2021 and watered once a week after making sure the soil was dry because rosemary plants do not tolerate wet soil. They were planted in an isolated region to prevent cross-hybridization. We used a drip irrigation system with water from aquaculture ponds. This water was rich in organic and mineral matter, which included essential nutrients such as nitrogen, phosphorus, and potassium and other micronutrients. These nutrients significantly enhance soil fertility, ensuring plant health and promoting vigorous growth (Zajdband, 2011; Hasimuna et al., 2023; Ignowski et al., 2023).^4^

### 2.2. Essential oil extraction procedure

Leaves of *M. × piperita* and *S. rosmarinus* were collected in July 2022 and dried in a dark, well-ventilated chamber. Once adequately dried, the leaves underwent essential oil distillation in October 2022. Using steam distillation, the essential oil was extracted from the leaves of both *M. × piperita* and *S. rosmarinus*. Briefly, the dried plant parts were placed in an alembic distillation unit (BF50 50L, Biofleur, Algeria). Steam was passed through the material for 3 h and the distillate was collected in a graduated cylinder. The oily layer was separated from the aqueous layer using a separating funnel, dried over anhydrous sodium sulfate, and stored at −4 °C until further analysis. The essential oil yield was calculated as follows:


$$T\%=\left(\frac{m}{m_{0}}\right)\times 100$$

Here, T% is the content expressed in %, m is the mass in grams of essential oil recovered, and m_0_ is the initial mass of plant material in grams.

### 2.3. Determination of essential oil composition using GC-MS

We tested the samples via the Technical Platform of Physico-Chemical Analysis (PTAPC-CRAPC) of Laghouat in Algeria employing a GCMSQP2020 instrument (Shimadzu, Kyoto, Japan). This instrument was equipped with a fused Rxi-5ms capillary column (Phase: Crossbond, 5% diphenyl/95% dimethyl polysiloxane) with dimensions of 30 m × 0.25 mm and film thickness of 0.25 μm (Restek, Bellefonte, PA, USA). This column has a phase identical to HP-1ms, HP-1msUI, DB-1ms, DB-5ms, DB-1msUI, Ultra-1, VF-1ms, ZB-1, and ZB-1ms and is also considered equivalent to the USP G1, G2 and G38 phases. Diluted material (0.5 μL) in n-hexane was injected using a split ratio of 1:80. The injector and detector temperatures were set at 250 °C and 310 °C, respectively. The column temperature was initially fixed at 50 °C for 2 min and then raised to 310 °C at a rate of 3 °C/min, and then it was held at 310 °C for 2 min. The carrier gas employed was helium (99.995% purity) at a flow rate of 1 mL/min. The parameters for the mass spectrometer were as follows: ionization voltage of 70 eV, ion source temperature of 200 °C, and electron ionization mass spectra generated within the mass range of 45–600 m/z. The retention indices were determined using the following equation:


$$LRI=100\times \left(\frac{t_{x}-t_{n}}{t_{n+1}-t_{n}}\right)+100\times n$$

Here, LRI is the linear retention index, t_x_ is the retention time of the targeted component, t_n_ is the retention time of the C_n_ alkane (lower bound n-alkane), and t_n+1_ is the retention time of the C_n+1_ alkane (higher bound n-alkane). The n-alkane series in these analyses was (n–C7–C33).

### 2.4. Chemicals and reagents

CRL and p-nitrophenyl laurate (p-NPL) were acquired from Sigma-Aldrich (St. Louis, MO, USA). All other chemicals and solvents utilized were of analytical quality and were also procured from Sigma-Aldrich.

### 2.5. Evaluation of in vitro antioxidant activity

We performed free radical scavenging and ABTS inhibition tests to evaluate the in vitro antioxidant activity of the *M. × piperita* and *S. rosmarinus* essential oils.

#### 2.5.1. DPPH assay

Our spectrophotometric method, based on the free radical DPPH^•^, was performed with a Multiskan GO microplate reader (Thermo Fisher Scientific, Waltham, MA, USA) at 517 nm. Extracted samples were dissolved in ethanol at concentrations of 0.1 to 1 mg/L, 120 μL of the sample solution was mixed with 120 μL of DPPH solution, and the absorbance of the mixture was measured after 30 min of incubation at ambient temperature in darkness (Gulcin and Alwasel, 2023). The radical scavenging activity (I%) was calculated using the following formula:


$$I\%=\left(\frac{A_{0}-A_{1}}{A_{0}}\right)\times 100$$

Here, I% is the percentage of inhibition, A_0_ is the absorption of the DPPH solution without the essential oil, and A_1_ is the absorption of the solution with the essential oil.

Percentage data were utilized to construct a graph illustrating the variation in absorbance in relation to the concentration of the essential oil samples. This analysis was used to determine the IC_50_ values, representing the concentration of essential oil (mg/mL) required to inhibit 50% of the free radicals (DPPH). Trolox and quercetin were employed as reference antioxidant standards for comparisons.

#### 2.5.2. ABTS assay

An ABTS radical cation solution (100 μL of ABTS, 15 μL of hydrogen peroxide, and 100 μL of peroxidase enzyme, with the volume completed to 100 mL with water) was prepared. For the measurement of the radical scavenging activity, 200 μL of diluted ABTS radical cation solution was mixed with 40 μL of a sample solution of essential oils at different dilutions (0.1 to 1 mg/mL), and the absorption was measured with the Multiskan GO microplate reader after 6 min at 417 nm. A calibration curve with Trolox and quercetin standards was measured in the range of 0.1–1 mg/mL (Ilyasov et al., 2020). Values of I% were calculated as described for the DPPH assay.

### 2.6. *C. rugosa* lipase assay

The p-NPL substrate was initially dissolved in isopropanol at a concentration of 1 mM. Subsequently, it was diluted at a ratio of 1:10 (v/v) in a buffer solution containing gum arabic with the pH adjusted to 7. To assess the lipase inhibitory activity, volumes of 20 μL of successive essential oil dilutions (0.1 to 1 mg/mL) were preincubated with 20 μL of enzyme solution (1 mg/mL) for 15 min at 37 °C. Subsequently, 180 μL of the p-NPL substrate was introduced. The reaction mixture was incubated for an additional 15 min at 37 °C. The absorbance of p-nitrophenol, indicated by the yellow color arising from the reaction, was promptly measured using the Multiskan GO microplate reader at 405 nm. For the negative control, the extract was replaced by an equivalent volume of methanol. Positive controls were employed to evaluate lipase activity in the presence and absence of the inhibitor. The percentage of lipase inhibition (I%) was calculated, followed by the determination of IC_50_ values. These results were then compared to results for orlistat and quercetin, which were used as reference compounds. The percentage inhibition of lipase was determined as follows:


$$Inhibition\%=\left(1-\frac{A-A_{i}}{A_{0}}\right)\times 100$$

Here, A is the absorbance of the sample containing essential oil, A_1_ is the absorbance of the sample devoid of lipase (i.e., the blank), and A_0_ is the absorbance of the control without essential oil. The 50% inhibitory concentration (IC_50_) was determined from the curve generated by graphing the percentage of inhibition against the final concentrations of essential oil (Lante et al., 2004).

### 2.7. Computational analysis

#### 2.7.1. Biological activity prediction (PASS)

PASS (“Prediction of Activity Spectra for Substances”) is structure–activity relationship software that provides ligand-based virtual screening to find ligands that bind to different receptors and/or molecules with certain biological activities (Abdou et al., 2017; Stasevych et al., 2017; Yildirim et al., 2017; James and Ramanathan, 2018; Pogodin et al., 2018). We used the PASS web server^5^ to identify the key compounds within *M. × piperita* and *S. rosmarinus* essential oils that could serve as potential inhibitors of CRL. The predictive analysis used the structural molecular formulae (SMILES) obtained from the PubChem database.^6^ The outcome of the PASS prediction was a ranked list of potential biological activities, organized in decreasing order of Pa – Pi values, where Pa represents the likelihood of classification as “active” and Pi denotes the chance of classification as “inactive.” We defaulted to Pa > Pi as the threshold for differentiating between “active” and “inactive” molecules. PASS-based virtual screening for a chemical library thus yields a compilation of molecules identified as “active,” or suitable for biological evaluation (Filimonov et al., 2014; Pogodin et al., 2018).

#### 2.7.2. ADMET evaluation

ADMET, which stands for “absorption, distribution, metabolism, excretion, and toxicity,” is a critical framework in drug research. Many drug candidates have failed during clinical trials or advanced stages of drug development due to inadequate efficacy or the presence of adverse side effects, underscoring the importance of evaluating these pharmacokinetic and toxicological properties early in the drug discovery process (Lucas et al., 2019). We evaluated the ADMET properties of the principal constituents of *M. × piperita* and *S. rosmarinus* essential oils using the SwissADME,^7^ ADMETlab 2.0 (Xiong et al., 2021),^8^ and admetSAR 3.0^9^ online servers. Quantitative predictions were performed using a set of rules that defined the molecular chemical structure’s ADMET characteristics based on available drug data (Alsawalha et al., 2019).

#### 2.7.3. Molecular docking

The studied compounds, including menthol, camphene, and orlistat, were obtained from the PubChem database.^10^ For assembly, we utilized Discovery Studio Visualizer Version 2024.^11^ PDB files for CRL (PDB ID: 1CRL), with resolution of 2.06 Å, and human pancreatic lipase (PDB ID: 1LPB), with resolution of 2.46 Å, were downloaded from the Protein Data Bank.^12^ We used AutoDock Tools Version 1.5.7 to prepare the enzymes for docking investigations, eliminating all water molecules, heteroatoms, cocrystallized solvents, and ligands. The polar hydrogens and partial charges were included in the structures (Morris et al., 2008; Valdés-Tresanco et al., 2020). Docking simulations were conducted using AutoDock Vina software (Trott and Olson, 2009; Di Muzio et al., 2017). The box’s center was established and the docking box was shown and delineated with AutoDock Tools. The docking was conducted blindly using a grid box of 72 × 68 × 68, with grid points spaced apart at 1 Å, concentrated at the protein locations (x = 72.33; y = 55.391; z = −20.425). All default parameters were employed. Fifty runs were conducted. The seed being searched was arbitrary. The favored conformations were those with the lowest binding energy inside the active site. The ratios of repetitions of the optimal solutions were established. The obtained docking findings were directly imported into Discovery Studio Visualizer Version 2024 (Serseg and Benarous, 2018; Serseg et al., 2020; Eberhardt et al., 2021).

### 2.8. Statistical analysis

Inhibitory activity was evaluated in all experiments, all of which were performed at least in triplicate, and results were presented as mean values with standard deviations. Data were analyzed at a significance level of p < 0.05. All results were generated using Microsoft Office Excel 2024.

## 3. Results and discussion

### 3.1. Extraction yield and composition of essential oils

The yield of peppermint was 1.81% in this study, Abbas et al. (2024) reported a yield of 0.2% for peppermint essential oil in Pakistan (District Khushab, Punjab), while in Ordu, Türkiye, the obtained yield of peppermint essential oil ranged from 0.72% to 1.82% (Saka et al., 2024).

The yield of the rosemary samples was 1.6% in this study, a value comparable to that obtained in the municipality of Bibans in the Algerian province of Bordj Bouarreridj (Annemer et al., 2022). The extraction yield of rosemary essential oil from plants cultivated in the Laghouat region in the present study was higher compared to plants cultivated in other regions of Algeria, with yields of 1.1% from the municipality of Maadid in eastern M’Sila and 0.7% in the province of Oran (Ain Turk) (Hendel et al., 2019). In Morocco, essential oil from plants of the Fez area was obtained with a yield of 1.4% (Elyemni et al., 2019), while plants from Oujda provided yields ranging from 0.6% to 1.7% (Sabbahi et al., 2020). The yield of essential oils can vary depending on several factors, such as the variety and type of plant material, maturity stage, and extraction conditions, all of which affect essential oil production (Abbas et al., 2022).

**Figure 1:**
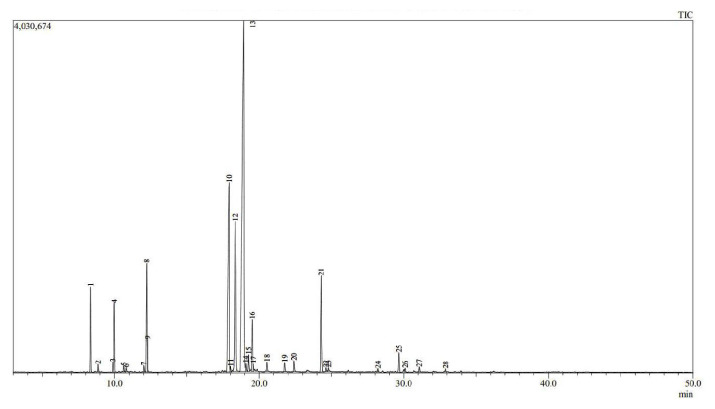
GC-MS chromatogram of *M. × piperita* essential oil.

**Figure 2:**
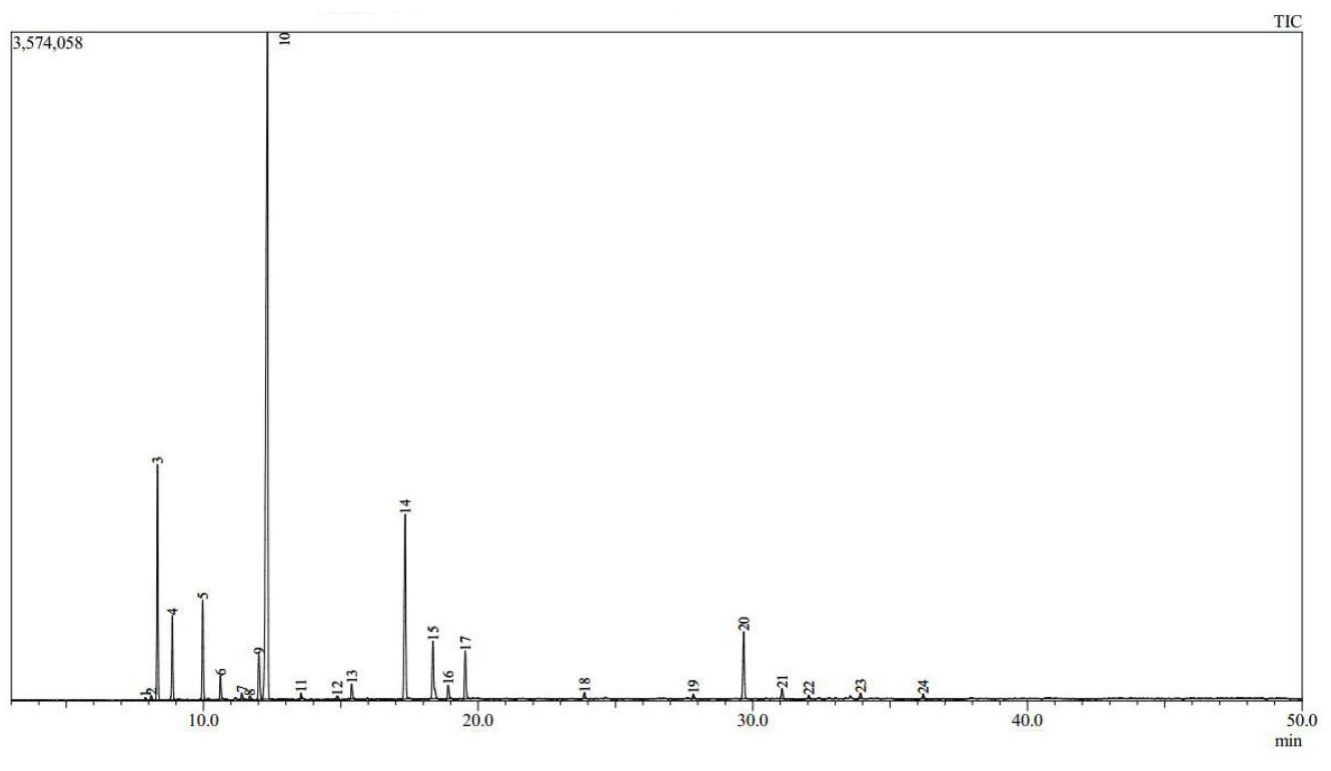
GC-MS chromatogram of *S. rosmarinus* essential oil.

We used GC-MS to analyze the chemical composition of the essential oils. Based on the results presented in Figures 1 and 2 for both essential oils, the relevant molecules were identified, along with their percentages and retention indices. For both plant species, we identified 100% of the compounds (Table 1).

The nine major compounds of the essential oil of *M. × piperita* were menthol (42.73%), menthone (16.43%), isoborneol (13.13%), limonene (5.33%), menthyl acetate (5.17%), α-pinene (3.39%), β-pinene (2.92%), isomenthol (2.63%), and eucalyptol (1.22%). A comparison with the literature data revealed quantitative and qualitative discrepancies between the chemical composition of *M. × piperita* essential oil from the Laghouat region and those obtained in other areas of Algeria. The essential oil of *M. × piperita* from Chiffa, Algeria, contained some of these compounds, including 32.93% menthol, 24.41% menthone, 8.08% cis-carane, and 7.89% eucalyptol (1,8-cineole) (Benzaid et al., 2019). In the National Park of El-Kala in El-Tarf, Algeria, the essential oil composition was characterized by menthol (49.89%), menthone (20.84%), isomenthone (7.25%), and 1,8-cineole (6.73%) (Benabdallah et al., 2018). The study conducted by Abbas et al. (2024) in Soon Valley, District Khushab, Punjab, Pakistan, corroborated these results with quantity fluctuations being observed, particularly for menthol (52.85%) and menthone (25.93%). The results also varied for the Ouezzane region of Morocco, where the major components were pulegone (17.56%), mintlactone (10.62%), D-carvone (9.24%), eucalyptol (7.53%), and thymol (6.06%) (El Omari et al., 2024).

The major compounds in the essential oil of *S. rosmarinus* were eucalyptol (1,8-cineole) (51.6%), camphor (11.28%), α-pinene (10.16%), sabinene (4.62%), caryophyllene (3.88%), camphene (3.74%), and borneol (3.66%). Another recently published study similarly revealed the presence of 1,8-cineole (28.6%–51.1%), α-pinene (9.9%–16.2%), camphor (5.3%–16.8%), β-pinene (2.2%–8.0%), and camphene (2.3%–7.7%) for all samples during all periods in essential oil obtained from *S. rosmarinus* (Annemer et al., 2022). Another study indicated that the major compounds of *S. rosmarinus* were 1,8-cineole (60.1% and 53.1 %), linalool (9.6% and 5.7%), α-terpineol (4.8% and 4.6%), camphor (1.1% and 11.6%), α-pinene (5.8% and 7.1%), and β-pinene (4.2% and 3.7%) from the Béchar region of Algeria, while camphor (23.2% and 22.9%), 1,8-cineole (13.2% and 14.4%), borneol (10.5% and 8.8%), verbenone (7.6% and 10.2%), campholenol (1.3% and 1.4%), α-pinene (10.0% and 9.0%), and camphene (4.7% and 3.2%) were reported from the essential oil of *S. rosmarinus* from the Adrar region (Bekhechi et al., 2024). In northern India, the major constituents of this essential oil were camphor (23.9%–35.8%), 1,8-cineole (18.0%–23.9%), α-pinene (4.5%–14.4%), verbenone (6.5%–12.4%), camphene (2.5%–6.9%), limonene (2.1%–2.8%), bornyl acetate (1.1%–4.1%), α-terpineol (1.9%–3.6%), and β-pinene (2.1%–3.3%) (Verma et al., 2020).

The numbers of components of oils fluctuate according to the isolation technique employed. The chemical compositions of *M. × piperita* and *S. rosmarinus* are particularly sensitive. According to the literature, several factors influence the quality and quantity of oil components, including environmental conditions (Li et al., 2020), the extraction process (Sadeh et al., 2019), the collection site (Riabov et al., 2020), the plant’s genotype (Ben Ayed et al., 2022), and the harvest season and stage (Li et al., 2020; Verma et al., 2020).

### 3.2. Antioxidant activity

We evaluated the antioxidant activities of *M. × piperita* and *S. rosmarinus* essential oils using the DPPH and ABTS radical scavenging assays and compared the results to those of quercetin and Trolox as positive controls (Table 2). Both essential oils exhibited significant antioxidant activity, as indicated by their IC_50_ values, which were comparable to those of the positive controls (Figure S1). Specifically, *M. × piperita* had IC_50_ values of 67 μg/mL for DPPH and 61 μg/mL for ABTS, while *S. rosmarinus* had IC_50_ values of 73 μg/mL and 63 μg/mL for DPPH and ABTS, respectively. These results are in line with those of previous studies reporting the powerful antioxidant properties of *S. rosmarinus* and *M. × piperita* essential oils (Chraibi et al., 2020; El Omari et al., 2024). *M. × piperita* essential oil had IC_50_ values of 68 μg/mL for DPPH radical scavenging and 76 μg/mL for ABTS (El Omari et al., 2024). A different study showed that *S. rosmarinus* essential oil had strong antioxidant properties due to its composition involving various phenolic aromatic rings, with an IC_50_ value of 2770 μg/mL for DPPH radical scavenging (Chraibi et al., 2020). These findings suggest that the essential oils of *M. × piperita* and *S. rosmarinus* could serve as natural sources of antioxidants for various applications, including food preservation, medicine manufacturing, and cosmetic formulations (Diniz do Nascimento et al., 2020).

### 3.3. Antilipase assay

We evaluated the inhibitory effects of peppermint and rosemary essential oils in comparison to quercetin and orlistat to quantify their enzymatic activities against CRL. IC_50_ values were used to determine the inhibitory potency of these extracts, representing the concentration required to inhibit 50% of the enzyme’s activity. The results are presented in Table 3 (Figure S1).

Table 3 reveals that all tested extracts exerted antilipase activity. *M. × piperita* essential oil, *S. rosmarinus* essential oil, quercetin, and orlistat had IC_50_ values of 0.56 ± 0.005, 0.69 ± 0.008, 0.32 ± 0.004, and 0.06 ± 0.000 mg/mL, respectively. These results confirm the potent inhibitory activity of both peppermint and rosemary essential oils against CRL. Compared to quercetin, these essential oils can be considered among the most effective natural medicines for treating obesity today. The impact of the essential oils investigated here against CRL has not been previously reported in the literature except for one previous study in which peppermint essential oil demonstrated weak lipase inhibitory effects compared to our findings, with an IC_50_ value of 1.09 mg/mL (Serseg et al., 2018). In a study of the inhibitory effect of *S. rosmarinus* essential oil against pancreatic lipase enzyme, the IC_50_ value was 0.03225 mg/mL, confirming the importance of *S. rosmarinus* essential oil in treating obesity (Ali et al., 2014). Other studies have tested the inhibition of other types of lipase using essential oils from taxa of the family Lamiaceae. Lante et al. (2004) conducted a study of the activity of oregano essential oil against two types of lipase and found that this essential oil had an IC_50_ value of 5.09 μg/mL against *Candida antarctica* lipase and an IC_50_ value of 7.26 μg/mL against *Pseudomonas fluorescens* lipase. Other research has examined the effects of essential oils from different plant families against CRL. These oils had IC_50_ values of 1.78 mg/mL for *Cinnamomum zeylanicum* (Lauraceae) and 1.13 mg/mL for *Syzygium aromaticum* (Myrtaceae) (Serseg et al., 2018).

### 3.4. Computational analysis

#### 3.4.1. Biological activity prediction (PASS)

To further explore the possible use of the powerful inhibitors found in *M. × piperita* and *S. rosmarinus* essential oils in combating obesity, we used the online PASS server to construct their biological activity profiles. This computer program forecasts possible biological activities utilizing an extensive library of chemical compounds and their corresponding activities. Table 4 and Table S1 present the anticipated effects on obesity in terms of weight reduction for the most efficacious inhibitors, offering insights into their prospective therapeutic significance in tackling this widespread health issue. Table 4 presents the list of identified compounds and their potential activities against obesity based on the degrees of potential activity (Pa) and potential inactivity (Pi). Higher Pa scores indicate that a compound is more likely to exhibit antiobesity activity, although Pa scores are not absolute predictors of efficacy. Rather, they indicate potential efficacy. Table 4 reveals several promising compounds for the treatment of obesity, including carvacrol, ρ-cymene and 3-octanol, with Pa scores as high as 0.689, 0.626, and 0.583, respectively. This suggests that these compounds could be further investigated as potential antiobesity agents. However, we must keep in mind that the efficacy of these compounds may be affected by their bioavailability, metabolism, and specific targets within the complex human biological system. Moving forward, it is necessary to study these compounds and validate their efficacy in terms of lipase inhibition, which we address in the following section on molecular docking, to confirm their applicability in obesity management.

#### 3.4.2. Molecular docking

We analyzed all constituents of the obtained *M. × piperita* and *S. rosmarinus* essential oils to investigate the interactions between inhibitors and lipase in silico. This study aimed to identify the key compounds that inhibit the lipase enzyme and obstruct the substrate from accessing the enzyme by examining pancreatic lipase inhibitors and CRL and comparing the results with the inhibition effects of orlistat against the same enzymes. The best docking pose for each inhibitor was determined based on the number of repeat types observed from generated solutions (Serseg et al., 2018). The docking results (Table 5) indicated that all molecules exhibited significant inhibitory effects depending on the number of solutions produced, thus confirming the in vitro results. The type of inhibition can be predicted based on interactions between the inhibitors and the amino acids of active sites, namely His449, Gly123, and Gly124 (Serseg and Benarous, 2018). Other amino acids were not directly involved in the process but were crucial for the stability of the ligand–enzyme complex, such as Phe344 (Manetti et al., 2000), the first amino acid of the tunnel cavity in CRL. The amino acids implicated in the active site of pancreatic lipase are Ser152 (Hadváry et al., 1991; Egloff et al., 1995), His263, and Phe77 (Nguyen et al., 2020).

We observed that the peppermint essential oil consisted mainly of menthol (42.73%), menthone (16.43%), menthyl acetate (5.17%), caryophyllene (1.07%), and α-humulene (0.27%), which interact with the active site of lipase with several hydrophobic interactions. The terpenes menthol and menthyl acetate have a hydroxyl group and an oxygen atom, which may act as acceptors or proton donors, thus forming hydrogen bonds with the amino acids of the active site of 1LPB Ser152 with lengths of 2.81 and 2.23 Å. In addition, the five terpenes menthol, menthone, menthyl acetate, caryophyllene, and α-humulene have 100% repetition ratios and contain aromatic rings capable of hydrophobic interactions of pi-sigma, alkyl, and pi-alkyl with the hydrophobic amino acids His263, Phe215, Phe77, and Tyr114. The affinity energy of each terpene was −6.8, −6.2, −6.8, −7.4, and −7.8 kcal/mol, respectively. Among these five terpenes, menthone, caryophyllene, and α-humulene achieved binding energies of −5, −6.5, and −6.7 kcal/mol, respectively. These terpenes also form hydrophobic interactions of the pi-sigma, alkyl, and pi-alkyl types with the Phe344 and His449 amino acids of the active site of 1CRL.

The four main terpenes of rosemary essential oil, including camphor, caryophyllene, α-copaene, and α-humulene, exhibited 100% repetition ratios in their interactions with the active sites of both lipases, suggesting a robust and consistent binding pattern. Their aromatic ring structures facilitated hydrophobic interactions with key amino acids. Three terpenes interacted with His263, Phe215, Phe77, and Tyr114. These interactions were of the pi-sigma, pi-alkyl, and pi-sigma types. Camphor engaged in alkyl interactions with Arg256, Ala259, Ala260, Leu264, and Ile78. These hydrophobic interactions further stabilized the inhibitor–enzyme complexes, enhancing the inhibitory potential. The terpene camphor exhibited hydrogenic interaction with Arg256, which has a longer length of 2.75 Å. Notably, the binding energies of the four terpenes were −5.6, −7.4, −7.9, and −7.8 kcal/mol, indicating strong affinity for the lipase active site. These terpenes are interesting because they had binding energies close to that of CRL, reaching −5.2, −6.5, −6.6, and −6.7 kcal/mol. This suggests that these terpenes can exert significant inhibitory effects against pancreatic and fungal lipases.

**Figure 3:**
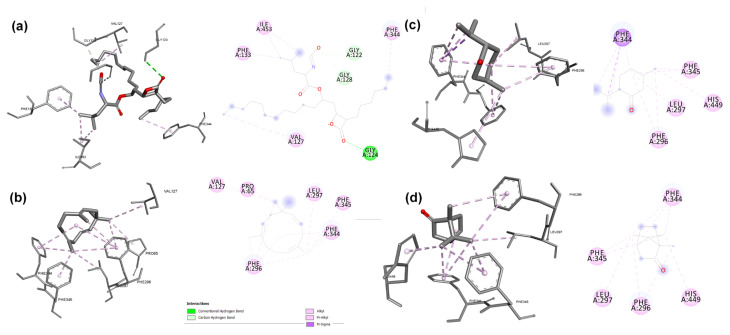
Molecular docking results for orlistat **(a)** and the most active terpenes of peppermint and rosemary essential oils, including α-humulene **(b)**, menthone **(c)**, and camphor **(d)**, against *Candida rugosa* lipase.

**Figure 4:**
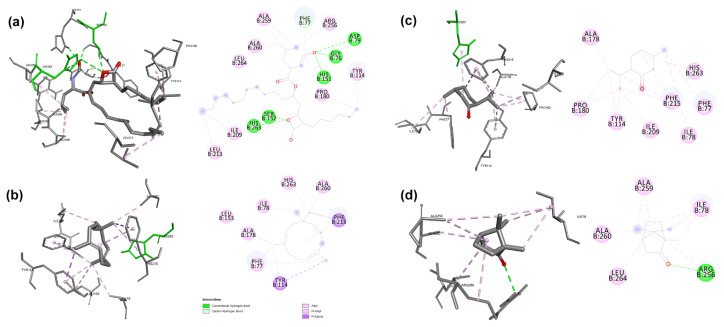
Molecular docking results for orlistat **(a)** and the most active terpenes of peppermint and rosemary essential oils, including α-humulene **(b)**, menthone **(c)**, and camphor **(d)**, against human pancreatic lipase.

Orlistat, a powerful drug used to treat obesity (Padwal et al., 2003), had hydrophobic interactions with the amino acid in the active site of 1CRL, Phe344, and the neighboring amino acids Gly128, Gly122, Ile453, Val127, and Phe133 with alkyl and pi-alkyl bonds (Figure 3). The amino acid of the active site, Gly124, forms hydrogen bonds with it, with length of 3.05 Å and estimated binding energy of −6.3 kcal/mol. It also had several interactions with the 1PLB enzyme (Figure 4), including hydrophobic interactions with several amino acids of the active site including Ala259, Ala260, Leu264, Arg256, and Tyr114, with alkyl and pi-alkyl bonds. The active site amino acids Ser152 and His263 exhibited hydrogen bonds with lengths of 2.16 and 3.08 Å, respectively. Orlistat had the same binding energy of −6.8 kcal/mol.

Overall, studying the different compounds of *M. × piperita* and *S. rosmarinus* essential oil showed that they could effectively inhibit lipase activity. They engaged with several amino acids in the enzyme active site by utilizing hydrogen bonds and hydrophobic interactions. These results provide significant insights into the molecular mechanisms that enable their functions as inhibitors, and they also have significant potential as therapeutic agents for the treatment of obesity and associated metabolic diseases.

#### 3.4.3. ADMET evaluations

ADMET studies were performed for the lipase inhibitors found in the *M. × piperita* and *S. rosmarinus* essential oils to determine whether they are safe for human use. We adopted a multipronged approach using online software, which provided initial insights into the potential ADMET properties of the key components. The results of all of these tests offered a full picture of the ADMET profiles of the inhibitors, allowing us to judge whether they could be used in therapeutic settings to treat obesity safely and effectively. The results are presented in Table 6, which shows how seven compounds of interest are absorbed, distributed, broken down, and eliminated and how dangerous they are. These compounds are menthol, menthone, methyl acetate, caryophyllene, camphor, α-copaene, α-methyl acetate, and α-humulene. The data indicated that all compounds had good oral absorption (>99%), with menthol acetate showing the highest value. Several compounds, including menthol, menthone, and camphor, exhibited excellent penetration of the blood–brain barrier, indicating potential central nervous system activity. Of note, α-copaene showed the highest binding to plasma protein, suggesting that it may have a longer half-life in the body. Interestingly, α-humulene blocked several cytochrome P450 enzymes, such as 2D6, 2C19, and 2C9. This suggests that the compound might interact with other drugs. This compound also had a lower clearance rate and longer half-life.

### 3.5. Conclusion

This study demonstrated that peppermint and rosemary essential oils possess significant capacity to inhibit the activity of lipase. This is interesting because it suggests that these essential oils may be useful in treating disorders associated with lipid metabolism and could be used as effective natural solutions to treat obesity. The results obtained from in silico studies confirmed that these essential oils are effective in inhibiting the lipase enzyme’s function with the possibility of occupation of the active site by their components.

## Supplementary materials

**Table S1:** Predicted antiobesity effects of the constituents of peppermint and rosemary essential oils.

N	Compounds	Pa	Pi	N	Compounds	Pa	Pi
**1**	Carvacrol	0.689	0.024	**24**	α -Thujene	0.431	0.102
**2**	ρ-Cymene	0.626	0.035	**25**	(Z)-Methyl cinnamate	0.425	0.106
**3**	3-Octanol	0.625	0.035	**26**	Camphor	0.421	0.108
**4**	Sabinene	0.583	0.044	**27**	neo-Verbanol	0.421	0.108
**5**	(Z)-Ethyl cinnamate	0.559	0.05	**28**	Terpinolene	0.411	0.114
**6**	Carvacrol, methyl ether	0.544	0.055	**29**	γ-Terpinene	0.41	0.115
**7**	Tricyclene	0.541	0.056	**30**	Verbenone	0.402	0.12
**8**	Terpinen-4-ol	0.54	0.056	**31**	α-Pinene	0.363	0.144
**9**	Isoborneol	0.538	0.056	**32**	Limonene	0.355	0.149
**10**	Borneol	0.538	0.056	**33**	Eucalyptol	0.345	0.155
**11**	α -Terpinene	0.535	0.057	**34**	γ-Muurolene	0.32	0.172
**12**	Heptanone <5-methyl-3->	0.529	0.059	**35**	α-Humulene	0.308	0.179
**13**	Menthol	0.514	0.064	**36**	Viridiflorene	0.308	0.18
**14**	Isomenthol	0.514	0.064	**37**	Caryophyllene	0.307	0.18
**15**	Menthyl acetate	0.512	0.065	**38**	(+)-delta-Cadinene	0.302	0.184
**16**	β-Pinene	0.501	0.069	**39**	Caryophyllenyl alcohol	0.266	0.213
**17**	Cymen-8-ol	0.498	0.07	**40**	Myrcene	0	0
**18**	Bornyl acetate	0.49	0.073	**41**	δ-Carene	0	0
**19**	Camphene	0.47	0.082	**42**	Linalool	0	0
**20**	ρ-Menth-1-en-9-ol acetate	0.466	0.084	**43**	Citronellal	0	0
**21**	Menthone	0.453	0.09	**44**	α-Terpineol	0	0
**22**	cis-Linalool oxide (pyranoid)	0.45	0.092	**45**	α-Copaene	0	0
**23**	Pulegone	0.445	0.094				

Figure S1 In vitro experimental results. (a) IC50 histogram of different extracts studied. (b) IC50 histogram of DPPH assay for *Mentha piperita* essential oil. (c) IC50 histogram of ABTS assay for *Mentha piperita* essential oil. (d) IC50 histogram of DPPH assay for *Salvia rosmarinus* essential oil. (e) IC50 histogram of ABTS assay for *Salvia rosmarinus* essential oil.

## Figures and Tables

**Table 1:** Chemical composition of essential oils of peppermint and rosemary.

Numbers	Retention index	Names of compounds	*M. piperita (*area%)	*S. rosmarinus (*area%)
1	914	Tricyclene	-	0.1
2	919	α -Thujene	-	0.18
3	924	α-Pinene	3.39	10.16
4	938	Heptanone <5-methyl-3->	0.33	-
5	938	Camphene	-	3.74
6	963	Sabinene	0.44	4.62
7	967	β-Pinene	2.92	1.11
8	983	Myrcene	0.27	-
9	988	3-Octanol	0.17	-
10	1003	δ-Carene	-	0.27
11	1009	α -Terpinene	-	0.23
12	1016	ρ-Cymene	0.28	2.65
13	1021	Limonene	5.33	-
14	1022	Eucalyptol	1.22	51.6
15	1051	γ-Terpinene	-	0.26
16	1080	Terpinolene	-	0.22
17	1092	Linalool	-	0.76
18	1134	Camphor	-	11.28
19	1147	Menthone	16.43	-
20	1148	Citronellal	0.27	-
21	1156	Isoborneol	13.13	-
22	1165	Borneol	-	3.66
23	1167	Menthol	42.73	
24	1168	Terpinen-4-ol	-	0.74
25	1172	cis-Linalool oxide (pyranoid)	0.46	-
26	1175	Cymen-8-ol	0.8	-
27	1181	Isomenthol	2.63	-
28	1181	α-Terpineol	-	2.65
29	1183	neo-Verbanol	0.35	-
30	1203	Verbenone	0.44	-
31	1230	Pulegone	0.47	-
32	1244	Carvacrol, methyl ether	0.55	-
33	1277	Bornyl acetate	-	0.32
34	1286	Menthyl acetate	5.17	-
35	1293	Carvacrol	0.21	-
36	1297	(Z)-Methyl cinnamate	0.16	-
37	1367	α-Copaene	-	0.24
38	1376	(Z)-Ethyl cinnamate	0.17	-
39	1410	Caryophyllene	1.07	3.88
40	1420	ρ-Menth-1-en-9-ol acetate	0.19	-
41	1444	α-Humulene	0.27	0.6
42	1468	γ-Muurolene	-	0.19
43	1488	Viridiflorene	0.17	-
44	1514	δ-Cadinene	-	0.29
45	1572	Caryophyllenyl alcohol	-	0.27
		Total	100%	100%

**Table 2:** IC_50_ values reflecting the antioxidant activities of *M. × piperita* and *S. rosmarinus* essential oils.

Essential oil	DPPH assay (μg/mL)	ABTS assay (μg/mL)
*M. piperita*	67±0.15	61±0.5
*S. rosmarinus*	73±0.3	63±0.2
Quercetin	7.3± 0.00	5.6±0.00
Trolox	5.8±0.00	0.95±0.00

**Table 3:** Inhibitory activities of *M. × piperita* and *S. rosmarinus* essential oils against *Candida rugosa* lipase.

Essential oil	IC_50_ value (mg/mL)
*M. piperita*	0.56±0.005
*S. rosmarinus*	0.69±0.008
Quercetin	0.32±0.004
Orlistat	0.06±0.000

**Table 4:** Predicted antiobesity effects of the constituents of peppermint and rosemary essential oils.

N	Compounds	Pa	Pi
**1**	Carvacrol	0.689	0.024
**2**	ρ-Cymene	0.626	0.035
**3**	3-Octanol	0.625	0.035
**4**	Sabinene	0.583	0.044
**5**	(Z)-Ethyl cinnamate	0.559	0.05
**6**	Carvacrol, methyl ether	0.544	0.055
**7**	Tricyclene	0.541	0.056
8	Terpinen-4-ol	0.54	0.056
**9**	Isoborneol	0.538	0.056
**10**	Borneol	0.538	0.056

**Table 5:** Molecular docking analysis of studied essential oil constituents against *Candida rugosa* lipase and human pancreatic lipase.

Enzyme-inhibitor	RR %	Energy (kcal/mol)	Closest residues	Hydrophobic interactions	Hydrogen bonds	Length (Å)
**Human pancreatic lipase (1LPB)**						
**Orlistat**	100	−6.8±0.00	Ala259, Ala260, Leu264, Arg256, Pro180, Ile209, Leu213, Tyr114	Alkyl, Pi-Alkyl	Gly76 His151 Ser152 His263 Asp79	2.625 2.481 2.168 3.088 3.346
**Menthol**	100	−6.8±0.00	Tyr114, Phe215, Pro180, Ile209, Phe77, His263	Pi-Sigma, Alkyl, Pi-Alkyl	Ser152 Phe215	2.814 2.277
**Menthone**	100	−6.2±0.00	Ala178, Ile78, Pro180, Ile209, Phe77, Tyr114, Phe215, His263	Alkyl, Pi-Alkyl	-	-
**Menthyl acetate**	100	−6.8±0.00	Ala178, Ala259, Ala260, Phe77, Tyr114, Phe215	Alkyl, Pi-Alkyl	Phe77 Ser152	2.507 2.237
**Caryophyllene**	100	−7.4±0.00	Phe215, Tyr114, Ala260, Ile78, Phe77, His263	Pi-Sigma, Alkyl, Pi-Alkyl	-	-
**Camphor**	100	−5.6±0.00	Arg256, Ala259, Ala260, Leu264, Ile78	Alkyl	Arg256	2.759
**α-Copaene**	100	−7.9±0.00	Tyr114, Ala178, Ala260, Leu153, Phe77, Phe215, His263	Pi-Sigma, Alkyl, Pi-Alkyl	-	-
**α-Humulene**	100	−7.8±0.00	Phe215, Tyr114, Ala178, Ala260, Ile78, Leu153, Phe77, His263	Pi-Sigma Alkyl Pi-Alkyl	-	-
***Candida rugosa*** ** lipase (1CRL)**						
**Orlistat**	100	−6.3±0.00	Gly128, Gly122, Ile453, Val127, Phe133, Phe344	Alkyl, Pi-Alkyl	Gly124	3.057
**α-Copaene**	100	−6.6±0.00	Leu297, Phe296, Phe344, Phe345, His449	Pi-Alkyl, Alkyl	-	-
**α-Humulene**	100	−6.7±0.00	Pro65, Val127, Leu297, Phe296, Phe344, Phe345	Alkyl, Pi-Alkyl	-	-
**Menthone**	80	−5.0±0.07	Phe344, Leu297, Phe296, Phe344, Phe345, His449	Pi-Sigma, Alkyl, Pi-Alkyl	-	-
**Caryophyllene**	80	−6.5±0.08	Pro65, Val127, Leu297, Phe296, Phe344, Phe345	Alkyl, Pi-Alkyl	-	-
**Camphor**	70	−5.2±0.14	Leu297, Phe296, Phe344, Phe345, His449	Alkyl, Pi-Alkyl	-	-

**Table 6:** Pharmacokinetic parameters of the constituents of the studied essential oils.

Pharmacokinetics	Menthol	Menthone	Menthyl acetate	Caryophyllene	Camphor	α-Copaene	α-Humulene
Molecular weight (Da)	156.150	154.140	198.160	204.190	152.120	204.19	204.190
**Absorption**							
Caco-2 cell permeability (nm/s)	0.8127	0.8127	0.8219	0.6327	0.8084	0.6705	0.6771
Human intestinal absorption (HIA %)	0.9944	0.9944	0.9970	0.9926	0.9971	1	0.9972
P-glycoprotein inhibition	None	None	None	None	None	None	None
Blood–brain barrier penetration (C.brain/C.blood)	0.9408	0.9408	0.9490	0.9536	0.983	0.9455	0.9733
MDCK cell permeability (nm/s)	2.7e-05	2.4e-05	2.2e-05	2.3e-05	2.2e-05	1.4e-05	1.6e-05
Plasma protein binding (%)	82.256	80.782	87.271	92.166	79.215	97.259	95.718
**Metabolism**							
Cytochrome P450 2D6 inhibition	None	None	None	None	None	None	Yes
Cytochrome P450 2D6 substrate	None	None	None	Yes	Yes	None	Yes
Cytochrome P450 3A4 inhibition	None	None	None	None	None	None	None
Cytochrome P450 3A4 substrate	None	None	None	None	None	None	None
Cytochrome P450 2C19 inhibition	None	None	None	None	None	None	Yes
Cytochrome P450 2C19 substrate	Yes	Yes	Yes	Yes	Yes	Yes	None
Cytochrome P450 2C9 inhibition	None	None	None	None	None	None	None
Cytochrome P450 2C9 substrate	Yes	Yes	Yes	Yes	Yes	None	Yes
**Excretion**							
Clearance	14.327	15.445	6.670	5.458	13.808	19.832	3.400
T1/2	0.354	0.585	0.318	0.100	0.701	0.059	0.403
**Toxicity**							
Ames test	None	None	None	None	None	None	None
Carcinogencity	None	Yes	None	None	None	None	None
HERG-inhibition	None	None	None	None	None	None	None
Eye Sensitization	Yes	Yes	Yes	Yes	Yes	None	Yes
Skin Sensitization	None	None	Yes	Yes	None	None	Yes
Hepatotoxicity	None	None	None	None	None	None	None
Respiratory toxicity	None	Yes	None	None	Yes	Yes	None

## Footnotes

1 World Health Organization (2024). Website https://www.who.int/news-room/fact-sheets/detail/obesity-and-overweight [accessed 25 December 2024].

2 Ministry of Tourism and Handicrafts (n.d.). Notre Wilaya (in French) [online]. Website https://laghouat.mta.gov.dz/fr/notre-wilaya/ [accessed 05 October 2024].

3 Britannica (2024). Laghouat [online]. Website https://www.britannica.com/place/Laghouat [accessed 05 October 2024].

4 Pullin R (n.d.). Integrated Agriculture-Aquaculture and the Environment [online]. Website https://www.fao.org/4/Y1187E/y1187e07.htm [accessed 06 October 2024].

5 PASS (2024). Website https://www.way2drug.com/passonline [accessed 20 September 2024].

6 PubChem (2024). Website https://pubchem.ncbi.nlm.nih.gov/ [accessed 19 September 2024].

7 SwissADME (2024). Website http://www.swissadme.ch/ [accessed 20 September 2024].

8 ADMETlab 2.0 (2024). Website https://admetmesh.scbdd.com/service/screening/index [accessed 20 September 2024].

9 admetSAR 3.0 (2024). Website https://lmmd.ecust.edu.cn/admetsar3/predict.php [accessed 20 September 2024].

10 PubChem (2024) Website. https://pubchem.ncbi.nlm.nih.gov/ [accessed 19 September 2024].

11 BIOVIA Discovery Studio 2024 Client (2024). Website https://biovia-discovery-studio-2024-client.software.informer.com/ [accessed 01 September 2024].

12 RCSB PDB: Homepage (2024). Website https://www.rcsb.org/ [accessed 19 September 2024].

## References

[b1-tjb-49-01-70] Abbas A Anwar F Ahmad N Rehman Atur Mohammed OA 2024 GC-MS analysis and nutra-pharmaceutical potential of *Mentha piperita* essential oil extracted by supercritical fluid extraction and hydro-distillation Heliyon 10 16 e35282 10.1016/J.HELIYON.2024.E35282 39220953 PMC11365357

[b2-tjb-49-01-70] Abbas A Anwar F Alqahtani SM Ahmad N Al-Mijalli SH 2022 Hydro-distilled and supercritical fluid extraction of eucalyptus camaldulensis essential oil: characterization of bioactives along with antioxidant, antimicrobial and antibiofilm activities Dose-Response 20 1 12 10.1177/15593258221125477 PMC9465602 36106059

[b3-tjb-49-01-70] Abdou WM Shaddy AA Kamel AA 2017 Structure-based design and synthesis of acyclic and substituted heterocyclic phosphonates linearly linked to thiazolobenzimidazoles as potent hydrophilic antineoplastic agents Chemical Papers 71 10 1961 1973 10.1007/s11696-017-0190-z

[b4-tjb-49-01-70] Ben Ali M Mnafgui K Feki A Damak M Allouche N 2014 In vitro antidiabetic, anti-obesity and antioxidant proprities of Rosemary extracts Journal of Advances in Chemistry 10 2 2305 2316 10.24297/JAC.V10I2.5497

[b5-tjb-49-01-70] Alsawalha M Bolla SR Kandakatla N Srinivasadesikan V Veeraraghavan VP 2019 Molecular docking and ADMET analysis of hydroxamic acids as HDAC2 inhibitors Bioinformation 15 6 380 10.6026/97320630015380 31312074 PMC6614126

[b6-tjb-49-01-70] Amaral-Machado L Oliveira WN Moreira-Oliveira SS Pereira DT Alencar ÉN 2020 Use of natural products in asthma treatment Evidence-based Complementary and Alternative Medicine 2020 10.1155/2020/1021258 PMC7040422 32104188

[b7-tjb-49-01-70] Annemer S Farah A Stambouli H Assouguem A Almutairi MH 2022 Chemometric investigation and antimicrobial activity of *Salvia rosmarinus* spenn essential oils Molecules 27 9 2914 10.3390/molecules27092914 35566267 PMC9099978

[b8-tjb-49-01-70] Bekhechi A Malti CEW Babali B Bouafia M Bekhechi C 2024 Chemical variability and anti-inflammatory activity of *Rosmarinus officinalis* L. leaf essential oil from Algerian Sahara Chemistry & Biodiversity 21 4 10.1002/CBDV.202302077 38388803

[b9-tjb-49-01-70] Ben Ayed R Moreau F Ben Hlima H Rebai A Ercisli S 2022 SNP discovery and structural insights into OeFAD2 unravelling high oleic/linoleic ratio in olive oil Computational and Structural Biotechnology Journal 20 1229 1243 10.1016/J.CSBJ.2022.02.028 35317231 PMC8914465

[b10-tjb-49-01-70] Benabdallah A Boumendjel M Aissi O Rahmoune C Boussaid M 2018 Chemical composition, antioxidant activity and acetylcholinesterase inhibitory of wild *Mentha* species from northeastern Algeria South African Journal of Botany 116 131 139 10.1016/J.SAJB.2018.03.002

[b11-tjb-49-01-70] Benzaid C Tichati L Djeribi R Rouabhia M 2019 Evaluation of the chemical composition, the antioxidant and antimicrobial activities of *Mentha*× *piperita* essential oil against microbial growth and biofilm formation Journal of Essential Oil Bearing Plants 22 2 335 346 10.1080/0972060X.2019.1622456

[b12-tjb-49-01-70] Birari RB Bhutani KK 2007 Pancreatic lipase inhibitors from natural sources: unexplored potential Drug Discovery Today 12 19–20 879 889 10.1016/J.DRUDIS.2007.07.024 17933690

[b13-tjb-49-01-70] Blüher M 2019 Obesity: global epidemiology and pathogenesis Nature Reviews Endocrinology 15 5 288 298 10.1038/S41574-019-0176-8 30814686

[b14-tjb-49-01-70] Boumeddiene MA Houyou Z Boutmedjet A Guibadj AN Benyahia MES 2023 Osmoprotection by proline accumulation induced by rainfall variations in three steppe plants species Aristida pungens, Retama raetam and Astragalus armatus under Mediterranean Arid bioclimate South Asian Journal of Experimental Biology 13 1 69 79 10.38150/SAJEB.13(1).P69-79

[b15-tjb-49-01-70] Boutabia L Telailia S Bouguetof I Guenadil F Chefrour A 2016 Composition chimique et activité antibactérienne des huile essentielles de *Rosmarinus officinalis* L. de la région de Hammamet (Tébessa-Algérie) Bulletin de la Societe Royale des Sciences de Liege 85 174 189 (in French). 10.25518/0037-9565.6050

[b16-tjb-49-01-70] Burbott AJ Hennessey JP Johnson WC Loomis WD 1983 Configuration of piperitone from oil of *Mentha piperita* Phytochemistry 22 10 2227 2230 10.1016/S0031-9422(00)80152-1

[b17-tjb-49-01-70] Chraibi M Farah A Elamin O Iraqui H Fikri-Benbrahim K 2020 Characterization, antioxidant, antimycobacterial, antimicrobial effcts of Moroccan rosemary essential oil, and its synergistic antimicrobial potential with carvacrol Journal of Advanced Pharmaceutical Technology and Research 11 1 25 29 10.4103/JAPTR.JAPTR_74_19 32154155 PMC7034174

[b18-tjb-49-01-70] Clark AM 1996 Natural products as a resource for new drugs Pharmaceutical Research 13 8 1133 1141 10.1023/A:1016091631721 8865302

[b19-tjb-49-01-70] Di Muzio E Toti D Polticelli F 2017 DockingApp: a user friendly interface for facilitated docking simulations with AutoDock Vina Journal of Computer-Aided Molecular Design 31 2 213 218 10.1007/S10822-016-0006-1 28063067

[b20-tjb-49-01-70] Diniz do Nascimento L de Moraes AAB da Costa KS Galúcio JMP Taube PS 2020 Bioactive natural compounds and antioxidant activity of essential oils from spice plants: new findings and potential applications Biomolecules 10 7 1 37 10.3390/BIOM10070988 PMC7407208 32630297

[b21-tjb-49-01-70] Eberhardt J Santos-Martins D Tillack AF Forli S 2021 AutoDock Vina 1.2.0: new docking methods, expanded force field, and python bindings Journal of Chemical Information and Modeling 61 8 3891 3898 10.1021/ACS.JCIM.1C00203 34278794 PMC10683950

[b22-tjb-49-01-70] Egloff MP Marguet F Buono G Verger R Cambillau C 1995 The 2, 46 Å resolution structure of the pancreatic lipase-colipase complex inhibited by a C11 alkyl phosphonate Biochemistry 34 9 2751 2762 10.1021/BI00009A003 7893686

[b23-tjb-49-01-70] El Omari N Chamkhi I Balahbib A Benali T Akhazzane M 2024 GC-MS-MS analysis and biological properties determination of *Mentha piperita* L., essential oils Biochemical Systematics and Ecology 116 104875 10.1016/J.BSE.2024.104875

[b24-tjb-49-01-70] Elyemni M Louaste B Nechad I Elkamli T Bauia A 2019 Extraction of essential oils of *Rosmarinus officinalis* L. by two different methods: hydrodistillation and microwave assisted hydrodistillation The Scientific World Journal 1 3659432 10.1155/2019/3659432 PMC6463580 31057339

[b25-tjb-49-01-70] Filimonov DA Lagunin AA Gloriozova TA Rudik AV Druzhilovskii DS 2014 Prediction of the biological activity spectra of organic compounds using the pass online web resource Chemistry of Heterocyclic Compounds 50 3 444 457 10.1007/S10593-014-1496-1

[b26-tjb-49-01-70] Gulcin İ Alwasel SH 2023 DPPH radical scavenging assay Processes 11 2248 10.3390/PR11082248

[b27-tjb-49-01-70] Hadváry P Sidler W Meister W Vetter W Wolfer H 1991 The lipase inhibitor tetrahydrolipstatin binds covalently to the putative active site serine of pancreatic lipase Journal of Biological Chemistry 266 4 2021 2027 10.1016/S0021-9258(18)52203-1 1899234

[b28-tjb-49-01-70] Hammond RA Levine R 2010 The economic impact of obesity in the United States Diabetes, Metabolic Syndrome and Obesity: Targets and Therapy 3 285 295 10.2147/DMSOTT.S7384 21437097 PMC3047996

[b29-tjb-49-01-70] Hannour K Boughdad A Maataoui A Bouchelta A 2018 Chemical composition of *Rosmarinus officinalis*(Lamiaceae) essential oils and evaluation of their toxicity against *Bruchus rufimanus*(Coleoptera: Chrysomelidae: Bruchinae) in Morocco International Journal of Tropical Insect Science 38 3 192 204 10.1017/S1742758418000012

[b30-tjb-49-01-70] Hasimuna OJ Maulu S Nawanzi K Lundu B Mphande J 2023 Integrated agriculture-aquaculture as an alternative to improving small-scale fish production in Zambia Frontiers in Sustainable Food Systems 7 1161121 10.3389/FSUFS.2023.1161121

[b31-tjb-49-01-70] Hendel N Napoli E Sarri M Saija A Cristani M 2019 Essential oil from aerial parts of wild Algerian rosemary: screening of chemical composition, antimicrobial and antioxidant activities Journal of Essential Oil Bearing Plants 22 1 1 17 10.1080/0972060X.2019.1590246

[b32-tjb-49-01-70] Ignowski L Belton B Ali H Thilsted SH 2023 Integrated aquatic and terrestrial food production enhances micronutrient and economic productivity for nutrition-sensitive food systems Nature Food 4 10 866 873 10.1038/s43016-023-00840-8 37666998 PMC10589083

[b33-tjb-49-01-70] Ilyasov IR Beloborodov VL Selivanova IA Terekhov RP 2020 ABTS/PP decolorization assay of antioxidant capacity reaction pathways International Journal of Molecular Sciences 21 1131 10.3390/IJMS21031131 32046308 PMC7037303

[b34-tjb-49-01-70] James N Ramanathan K 2018 Discovery of potent ALK inhibitors using pharmacophore-informatics strategy Cell Biochemistry and Biophysics 76 1–2 111 124 10.1007/S12013-017-0800-Y 28477056

[b35-tjb-49-01-70] Khalvandi M Amerian M Pirdashti H Keramati S Hosseini J 2019 Essential oil of peppermint in symbiotic relationship with *Piriformospora indica* and methyl jasmonate application under saline condition Industrial Crops and Products 127 195 202 10.1016/J.INDCROP.2018.10.072

[b36-tjb-49-01-70] Kopelman PG 2000 Obesity as a medical problem Nature 404 6778 635 643 10.1038/35007508 10766250

[b37-tjb-49-01-70] Kumar A Chauhan S 2021 Pancreatic lipase inhibitors: the road voyaged and successes Life Sciences 271 119115 10.1016/J.LFS.2021.119115 33515565

[b38-tjb-49-01-70] Lante A Crapisi A Lomolino G Spettoli P 2004 Chemical parameters, biologically active polyphenols and sensory characteristics of some Italian organic wines Journal of Wine Research 15 3 203 209 10.1080/09571260500142054

[b39-tjb-49-01-70] Lešnik S Furlan V Bren U 2021 Rosemary (*Rosmarinus officinalis* L.): extraction techniques, analytical methods and health-promoting biological effects Phytochemistry Reviews 20 6 1273 1328 10.1007/S11101-021-09745-5

[b40-tjb-49-01-70] Li Y Kong D Fu Y Sussman MR Wu H 2020 The effect of developmental and environmental factors on secondary metabolites in medicinal plants Plant Physiology and Biochemistry 148 80 89 10.1016/J.PLAPHY.2020.01.006 31951944

[b41-tjb-49-01-70] Lopez-Jimenez F Almahmeed W Bays H Cuevas A Di Angelantonio E 2022 Obesity and cardiovascular disease: mechanistic insights and management strategies. A joint position paper by the World Heart Federation and World Obesity Federation European Journal of Preventive Cardiology 29 17 2218 2237 10.1093/EURJPC/ZWAC187 36007112

[b42-tjb-49-01-70] Lucas AJ Sproston JL Barton P Riley RJ 2019 Estimating human ADME properties, pharmacokinetic parameters and likely clinical dose in drug discovery Expert Opinion on Drug Discovery 14 12 1313 1327 10.1080/17460441.2019.1660642 31538500

[b43-tjb-49-01-70] Lunagariya NA Patel NK Jagtap SC Bhutani KK 2014 Inhibitors of pancreatic lipase: state of the art and clinical perspectives EXCLI Journal 13 897 10.17877/DE290R-6941 26417311 PMC4464291

[b44-tjb-49-01-70] Manetti F Mileto D Corelli F Soro S Palocci C 2000 Design and realization of a tailor-made enzyme to modify the molecular recognition of 2-arylpropionic esters by *Candida rugosa* lipase Biochimica et Biophysica Acta (BBA) - Protein Structure and Molecular Enzymology 1543 1 146 158 10.1016/S0167-4838(00)00185-0 11087950

[b45-tjb-49-01-70] McKay DL Blumberg JB 2006 A review of the bioactivity and potential health benefits of peppermint tea (*Mentha piperita* L.) Phytotherapy Research 20 8 619 633 10.1002/PTR.1936 16767798

[b46-tjb-49-01-70] Miyake K 2001 Pancreatic Lipase. Rinsho byori The Japanese Journal of Clinical Pathology Suppl 116 61 79 11797385

[b47-tjb-49-01-70] Morris GM Huey R Olson AJ 2008 Using AutoDock for ligand-receptor docking Current Protocols in Bioinformatics SUPPL 24 10.1002/0471250953.BI0814S24 19085980

[b48-tjb-49-01-70] Najmi A Javed SA Al Bratty M Alhazmi HA 2022 Modern Approaches in the discovery and development of plant-based natural products and their analogues as potential therapeutic agents Molecules 27 2 10.3390/MOLECULES27020349 PMC8779633 35056662

[b49-tjb-49-01-70] Nguyen PTV Huynh HA van Truong D Tran TD Vo CVT 2020 Exploring aurone derivatives as potential human pancreatic lipase inhibitors through molecular docking and molecular dynamics simulations Molecules 25 20 4657 10.3390/MOLECULES25204657 33066044 PMC7587340

[b50-tjb-49-01-70] Okunogbe A Nugent R Spencer G Ralston J Wilding J 2021 Economic impacts of overweight and obesity: current and future estimates for eight countries BMJ Global Health 6 10 10.1136/BMJGH-2021-006351 PMC8487190 34737167

[b51-tjb-49-01-70] Padwal R Li SK Lau DCW 2003 Long-term pharmacotherapy for obesity and overweight Cochrane Database of Systematic Reviews 4 CD004094 10.1002/14651858.CD004094.pub2 14584004

[b52-tjb-49-01-70] Pogodin PV Lagunin AA Rudik AV Filimonov DA Druzhilovskiy DS 2018 How to achieve better results using PASS-based virtual screening: Case study for kinase inhibitors Frontiers in Chemistry 6 APR 10.3389/FCHEM.2018.00133 PMC5935003 29755970

[b53-tjb-49-01-70] Riabov PA Micić D Božović RB Jovanović DV Tomić A 2020 The chemical, biological and thermal characteristics and gastronomical perspectives of *Laurus nobilis* essential oil from different geographical origin Industrial Crops and Products 151 112498 10.1016/J.INDCROP.2020.112498

[b54-tjb-49-01-70] Rocha S Rufino AT Freitas M Silva AMS Carvalho F 2023 Methodologies for assessing pancreatic lipase catalytic activity: a review Critical Reviews in Analytical Chemistry 54 8 3038 3065 10.1080/10408347.2023.2221731 37335098

[b55-tjb-49-01-70] Sabbahi M ElHassouni A Tahani A El Bachin A 2020 Altitude effect on the chemical composition and antioxidant activity of rosemary in the region of Talsint (Morocco) Moroccan Journal of Chemistry 8 4 866 875 10.48317/IMIST.PRSM/MORJCHEM-V8I4.18311

[b56-tjb-49-01-70] Sadeh D Nitzan N Chaimovitsh D Shachter A Ghanim M 2019 Interactive effects of genotype, seasonality and extraction method on chemical compositions and yield of essential oil from rosemary (*Rosmarinus officinalis* L.) Industrial Crops and Products 138 111419 10.1016/J.INDCROP.2019.05.068

[b57-tjb-49-01-70] Safaei M Sundararajan EA Driss M Boulila W Shapi’i A 2021 A systematic literature review on obesity: understanding the causes & consequences of obesity and reviewing various machine learning approaches used to predict obesity Computers in Biology and Medicine 136 104754 10.1016/J.COMPBIOMED.2021.104754 34426171

[b58-tjb-49-01-70] Saka AK Uğur A Açıkgöz MA 2024 Assessment of agronomic traits and essential oil yield in various mint (*Mentha piperita* L.) genotypes Akademik Ziraat Dergisi 13 1 1 12 10.29278/azd.1465733

[b59-tjb-49-01-70] Sauter ER 2020 Cancer prevention and treatment using combination therapy with natural compounds Expert Review of Clinical Pharmacology 13 3 265 285 10.1080/17512433.2020.1738218 32154753

[b60-tjb-49-01-70] Serseg T Benarous K 2018 The inhibitory effect of some drugs on *Candida rugosa* lipase and human pancreatic lipase: in vitro and in silico studies Endocrine, Metabolic & Immune Disorders - Drug Targets 18 6 602 609 10.2174/1871530318666180319093342 29557755

[b61-tjb-49-01-70] Serseg T Benarous K Lamrani M Yousfi M 2020 Lepidine B from *Lepidium sativum* seeds as multi-functional anti-Alzheimer’s disease agent: in vitro and in silico studies Current Computer-Aided Drug Design 17 3 360 377 10.2174/1573409916666200302120305 32116197

[b62-tjb-49-01-70] Serseg T Benarous K Yousfi M 2018 The inhibitory effect of three essential oils on *Candida rugosa* lipase: in vitro and in silico studies The Natural Products Journal 10 3 208 215 10.2174/2210315508666181009112415

[b63-tjb-49-01-70] Soliman FM El-Kashoury EA Fathy MM Gonaid MH 1994 Analysis and biological activity of the essential oil of *Rosmarinus officinalis* L. from Egypt Flavour and Fragrance Journal 9 1 29 33 10.1002/ffj.2730090107

[b64-tjb-49-01-70] Stasevych M Zvarych V Lunin V Deniz NG Gokmen Z 2017 Computer-aided prediction and cytotoxicity evaluation of dithiocarbamates of 9, 10-anthracenedione as new anticancer agents SAR and QSAR in Environmental Research 28 5 355 366 10.1080/1062936X.2017.1323796 28524703

[b65-tjb-49-01-70] Trott O Olson AJ 2009 AutoDock Vina: improving the speed and accuracy of docking with a new scoring function, efficient optimization, and multithreading Journal of Computational Chemistry 31 2 455 461 10.1002/JCC.21334 PMC3041641 19499576

[b66-tjb-49-01-70] Valdés-Tresanco MS Valdés-Tresanco ME Valiente PA Moreno E 2020 AMDock: a versatile graphical tool for assisting molecular docking with Autodock Vina and Autodock4 Biology Direct 15 1 12 10.1186/S13062-020-00267-2 32938494 PMC7493944

[b67-tjb-49-01-70] Verma RS Padalia RC Chauhan A Upadhyay RK Singh VR 2020 Productivity and essential oil composition of rosemary (*Rosmarinus officinalis* L.) harvested at different growth stages under the subtropical region of north India Journal of Essential Oil Research 32 2 144 149 10.1080/10412905.2019.1684391

[b68-tjb-49-01-70] Xiong G Wu Z Yi J Fu L Yang Z 2021 ADMETlab 2,0: an integrated online platform for accurate and comprehensive predictions of ADMET properties Nucleic Acids Research 49 W1 W5 W14 10.1093/nar/gkab255 33893803 PMC8262709

[b69-tjb-49-01-70] Yıldırım H Bayrak N Tuyun AF Kara EM Çelik BÖ 2017 2,3-Disubstituted-1,4-naphthoquinones containing an arylamine with trifluoromethyl group: synthesis, biological evaluation, and computational study RSC Advances 7 41 25753 25764 10.1039/C7RA00868F

[b70-tjb-49-01-70] Zajdband AD 2011 Integrated agri-aquaculture systems Lichtfouse E Genetics, Biofuels and Local Farming Systems Sustainable Agriculture Reviews 7 Dordrecht, the Netherlands Springer 87 127 10.1007/978-94-007-1521-9_4

[b71-tjb-49-01-70] Zhang L Song J Kong L Yuan T Li W 2020 The strategies and techniques of drug discovery from natural products Pharmacology and Therapeutics 216 107686 10.1016/J.PHARMTHERA.2020.107686 32961262

