# Supplementary figures and images for: Antilipase activities of cultivated peppermint and rosemary essential oils: in vitro and in silico studies
Source: Turk J Biol. 2025 Jan 14;49(1):70–84. doi: 10.55730/1300-0152.2725 (PMC11913368; doi:10.55730/1300-0152.2725)

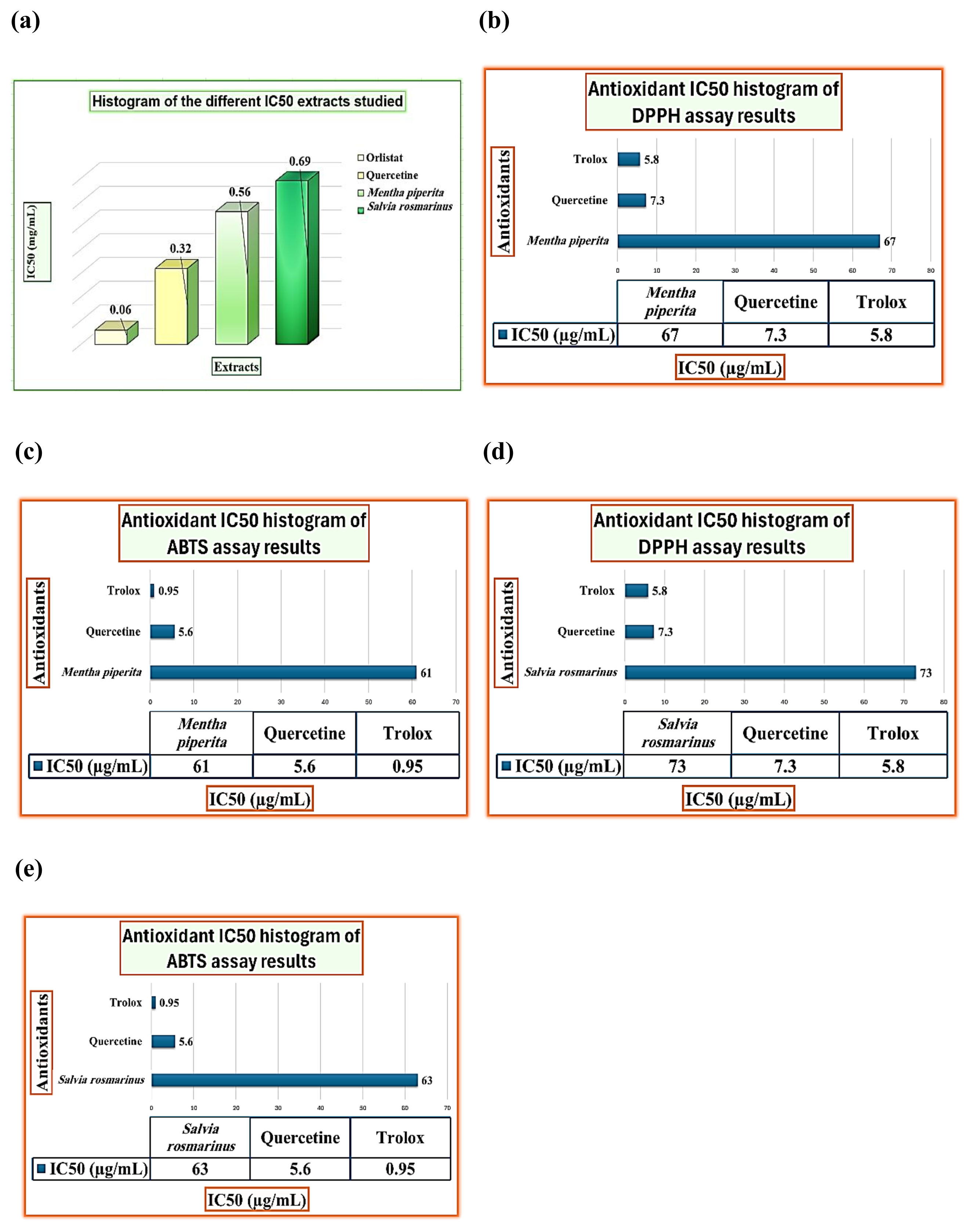

Supplement: Figure S1 — In vitro experimental results. (a) IC50 histogram of different extracts studied. (b) IC50 histogram of DPPH assay for Mentha piperita essential oil. (c) IC50 histogram of ABTS assay for Mentha piperita essential oil. (d) IC50 histogram of DPPH assay for Salvia rosmarinus essential oil. (e) IC50 histogram of ABTS assay for Salvia rosmarinus essential oil. [file tjb-49-01-70s1.tif]
